# A fine recognition method of strawberry ripeness combining Mask R-CNN and region segmentation

**DOI:** 10.3389/fpls.2023.1211830

**Published:** 2023-07-28

**Authors:** Can Tang, Du Chen, Xin Wang, Xindong Ni, Yehong Liu, Yihao Liu, Xu Mao, Shumao Wang

**Affiliations:** ^1^ College of Engineering, China Agricultural University, Beijing, China; ^2^ State Key Laboratory of Intelligent Agricultural Power Equipment, Henan, China; ^3^ Beijing Key Laboratory of Optimized Design for Modern Agricultural Equipment, Beijing, China

**Keywords:** strawberry, ripeness recognition, deep learning, image processing, Mask R-CNN

## Abstract

As a fruit with high economic value, strawberry has a short ripeness period, and harvesting at an incorrect time will seriously affect the quality of strawberries, thereby reducing economic benefits. Therefore, the timing of its harvesting is very demanding. A fine ripeness recognition can provide more accurate crop information, and guide strawberry harvest management more timely and effectively. This study proposes a fine recognition method for field strawberry ripeness that combines deep learning and image processing. The method is divided into three stages: In the first stage, self-calibrated convolutions are added to the Mask R-CNN backbone network to improve the model performance, and then the model is used to extract the strawberry target in the image. In the second stage, the strawberry target is divided into four sub-regions by region segmentation method, and the color feature values of B, G, L, a and S channels are extracted for each sub-region. In the third stage, the strawberry ripeness is classified according to the color feature values and the results are visualized. Experimental results show that with the incorporation of self-calibrated convolutions into the Mask R-CNN, the model’s performance has been substantially enhanced, leading to increased robustness against diverse occlusion interferences. As a result, the final average precision (AP) has improved to 0.937, representing a significant increase of 0.039 compared to the previous version. The strawberry ripeness classification effect is the best on the SVM classifier, and the accuracy under the combined channel BGLaS reaches 0.866. The classification results are better than common manual feature extraction methods and AlexNet, ResNet18 models. In order to clarify the role of the region segmentation method, the contribution of different sub-regions to each ripeness is also explored. The comprehensive results demonstrate that the proposed method enables the evaluation of six distinct ripeness levels of strawberries in the complex field environment. This method can provide accurate decision support for strawberry refined planting management.

## 1 Introduction

Strawberries, being a typical non-climacteric fruit, can continue to ripen after being picked, but their edible quality does not improve with further ripening (Chen et al., 2014; Van de Poel et al., 2014). Once strawberries begin to bear fruit, they typically take 20-30 days to reach full ripeness. Furthermore, the transition from the white ripe stage to the fully ripe stage takes only about 7 days for strawberries. Therefore, an efficient and accurate method for assessing strawberry ripeness would align with practical requirements. The traditional manual observation method is characterized by low work efficiency, poor accuracy and significant variability, rendering it inadequate to meet the demands of efficient detection. Despite the high accuracy of the sensor detection method, its requirement for professional operation and low efficiency make it unsuitable for large-scale detection (Moghimi et al., 2010; Abbaszadeh et al., 2014; Aghilinategh et al., 2020). Therefore, it is of great significance to study an efficient and accurate strawberry ripeness judgment method in an unstructured environment for strawberry harvest management. However, the field environment where strawberries grow is characterized by leaf occlusion and fruit overlapping, presenting challenges in accurately recognizing the ripeness of strawberries.

With the advancement of new information technology and the promotion of technical methods, machine learning (ML) and deep learning (DL) have made significant strides in scene recognition and object classification. Considering their characteristics of faster detection, better generalization, and stronger robustness, these methods have also emerged as a research hotspot in strawberry detection and recognition (Yu et al., 2019; Pérez-Borrero et al., 2020; Le Louëdec and Cielniak, 2021). The current strawberry ripeness detection method predominantly revolve around the integration of ML, DL, and hyperspectral imaging techniques. Zhang et al. (2016) used PCA to obtain optimal wavelengths from hyperspectral images, and then extracted texture features from the optimal wavelength images. They finally obtained the best strawberry ripeness classification in SVM with the combined information of the best wavelength and texture features. Shao et al. (2020) extracted effective wavelengths for field and outdoor hyperspectral strawberry images, respectively. Finally, their PLS-DA and LS-SVM classifiers achieved between 91.7% and 96.7% accuracy in field strawberry ripeness classification. Su et al. (2021) established a 1D residual network and a 3D residual network to process 1D and 3D strawberry hyperspectral data. The accuracy of ripeness classification exceeded 84% in both networks. Raj et al. (2022) obtained over 98% ripeness classification accuracy when using the full spectrum data of strawberries as the input data of SVM. Furthermore, they developed a strawberry water content index based on a portion of the spectral data from the band, achieving the highest accuracy of 71.2% when using the water content index as input data. Additionally, there have been studies exploring the utilization of image processing techniques in conjunction with deep learning for strawberry ripeness detection. Fan et al. (2022) used a dark channel enhancement algorithm to preprocess strawberry images taken at night, and finally achieved a ripeness recognition accuracy of over 90% on YOLOv5. Despite achieving some results in strawberry ripeness estimation, hyperspectral imaging is known for its high cost and inconvenience in practical usage. Moreover, its application is primarily limited to indoor environments, making it challenging to fulfill the requirements of real-time detection in the field.

According to the characteristics of strawberry at different ripeness stages, most of the above studies have categorized strawberry ripeness into 2-3 levels. However, the classification of 2-3 levels is rough and cannot provide an accurate decision-making basis for strawberry harvesting management. On the one hand, foliar fertilizer spraying before strawberry ripening can increase the firmness of strawberries at harvest and prolong the storage time (He et al., 2018). This necessitates the identification of early ripeness in strawberries to determine optimal timing for fertilization. On the other hand, for the two different modes of on-site sales and off-site sales, it is necessary to identify the harvest ripeness of strawberries in the later stage to determine the harvest time. Therefore, considering the current large-scale strawberry cultivation, there is a need for finer ripeness grading to offer precise decision support for strawberry harvesting management.

Based on the above analysis, combined with deep learning technology and image processing technology, this paper proposes a strawberry ripeness recognition method combined with Mask R-CNN and region segmentation. This method not only enhances the segmentation accuracy of strawberries in complex field environments but also accurately estimates six distinct levels of ripeness, providing richer and more detailed information about strawberry maturation.

## 2 Materials and methods

### 2.1 Dataset

#### 2.1.1 Image acquisition

**Figure 1:**
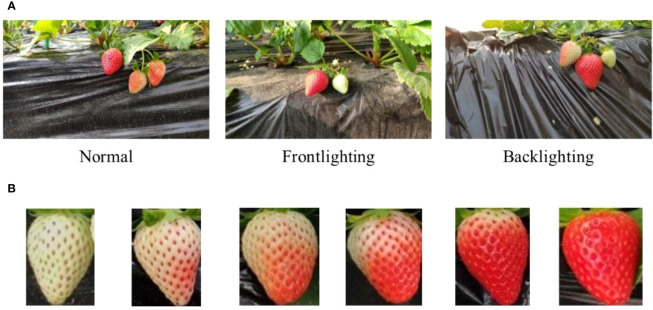
**(A)** Initial images. **(B)** Images of strawberries at different ripeness. From left to right: White, Breaking, Turning-1, Turning-2, Ripe and Full ripe.

In order to improve the robust performance of the model in various environments, the strawberry images for this study were acquired in two batches to increase data diversity. The first shot was taken on January 7, 2022 in a strawberry plantation in Changping District, Beijing, China, from 10:00 to 14:00, and the local weather was sunny and cloudless. The device used is an MI 8 smart mobile phone with a SONY IMX363 lens. The second shot was taken on February 9, 2023 in a strawberry plantation in Pinggu District, Beijing, China, from 13:00 to 17:00, and the local weather was cloudy. The device used is a MI 12X smart mobile phone, and the lens is SONY IMX766. The distance from the lens to the strawberry ridge was 0.2-0.3 m for each shooting, and finally 500 pictures with a size of 4032×2268 pixels and 700 pictures with a size of 4096×2304 pixels were obtained respectively. The pictures include images under different lighting conditions such as normal, frontlighting, and backlighting, as shown in **Figure 1A**. We compressed all images to a size of 1280×720 pixels to reduce computational cost.

#### 2.1.2 Dataset partitioning and annotation

The strawberry datasets were divided into two parts: instance segmentation dataset and image classification dataset. For the instance segmentation dataset, the initial images were randomly divided into 860 images for training set, 100 images for validation set, and 240 images for test set. Each strawberry contour was annotated with labelme annotation tool. For the image classification dataset, the dataset consisted of a series of strawberry patches. The training set comprised a total of 2172 strawberry patches, which were manually cropped from the training set of the instance segmentation task. The test set consisted of a total of 651 strawberry patches, which were detected by the instance segmentation model from the test set of the instance segmentation task.

Efficient and accurate decision-making is crucial for the management of large-scale strawberry harvesting in order to enhance economic benefits. This necessitates a more precise classification of strawberry ripeness to meet the requirements of the industry. Based on the physiological changes (Azodanlou et al., 2004; Zhang et al., 2011) and color representation of strawberries during the ripening process, the strawberry ripeness has been categorized into six levels: White, Breaking, Turning-1, Turning-2, Ripe and Full ripe. At White the fruit is light green, and it is basically no longer growing. At Breaking the fruit is one-fifth red and begins to enter the color changing period. It is suitable to apply foliar fertilizer to improve the hardness of the strawberry when it is mature. Turning-1 is two-fifths red strawberry, and Turning-2 is three-fifths red strawberry. At Ripe the strawberry is approximately four-fifths red, indicating it is ready for harvest, particularly for off-site sales. At Full ripe the strawberry is dark red and is completely ripe. Completely ripe strawberries offer the best taste but are not ideal for storage and transportation. Therefore, the Full ripe stage is considered the harvest period for local sales. The patches of strawberries with different ripeness are shown in **Figure 1B**. The details of the dataset are shown in **Table 1**.

**Table 1:** Strawberry ripeness classification dataset.

Ripeness category	#Training set	#Test set
White	603	178
Breaking	313	83
Turning-1	230	61
Turning-2	230	64
Ripe	359	116
Full ripe	437	149
Total	2172	651

### 2.2 Annotation validation

The strawberry ripeness labels are manually annotated, and the quality of the annotation results directly impacts the effectiveness of subsequent classification. Therefore, it is necessary to verify the accuracy of manual labels. Uniform Manifold Approximation and Projection for Dimension Reduction (UMAP) is a nonlinear data dimensionality reduction algorithm (McInnes et al., 2018). It can map the structural features of high-dimensional space *x*_i_ to low-dimensional space *y*_i_ for representation, and preserve the global structure of the data well. Through low-dimensional data visualization, potential relationships among raw data can be observed. We input the strawberry patches into UMAP for dimensionality reduction, and then observe the distribution of strawberries.

Let $\text{X}=\left\{x_{1},\;\ldots ,\;x_{N}\right\}$ be the input data set. First, we use the nearest neighbor or approximate nearest neighbor algorithm to obtain the k nearest neighbor set $\left\{x_{i1},\;\ldots ,\;x_{ik}\right\}$ , and then for each *x*_i_, we use Eq. (1) and (2) to find the nearest neighbor distance *ρ*_i_ and the normalization factor *σ*_i_.


(1) $$\rho_{i}=min\{d\left(x_{i},x_{ij}\right)|1\leq j\leq k,d\left(x_{i},x_{ij}\right)>0\}$$

(2) $$\sum_{j=1}^{k}exp\left(\frac{-max\left(0,d\left(x_{i},x_{ij}\right)-\rho_{i}\right)}{\sigma_{i}}\right)=log_{2}\left(k\right)$$

In high-dimensional space, the distance probability is expressed as Eq. (3) and (4).


(3) $$p_{i|j}=exp\left(\frac{-max\left(0,d\left(x_{i},x_{ij}\right)-\rho_{i}\right)}{\sigma_{i}}\right)$$

(4) $$p_{ij}=p_{i|j}+p_{j|i}-p_{i|j}p_{j|i}$$

In the low-dimensional space, the distance probability is expressed as Eq. (5), where *y*_i_, *y*_j_ are low-dimensional space data, a≈1.93, b≈0.79 are hyperparameters.


(5) $$q_{ij}={\left(1+a{\left(y_{i}-y_{j}\right)}^{2b}\right)}^{-1}$$

Finally, a low-dimensional representation of UMAP is obtained by minimizing the cross-entropy cost function, which can be expressed as Eq. (6).


(6) $$CE\left(X,Y\right)=\sum_{i}\sum_{j}[p_{ij}\left(X\right)log\left(\frac{p_{ij}\left(X\right)}{q_{ij}\left(Y\right)}\right)+\left(1-p_{ij}\left(X\right)\right)log\left(\frac{1-p_{ij}\left(X\right)}{1-q_{ij}\left(Y\right)}\right)]$$

**Figure 2:**
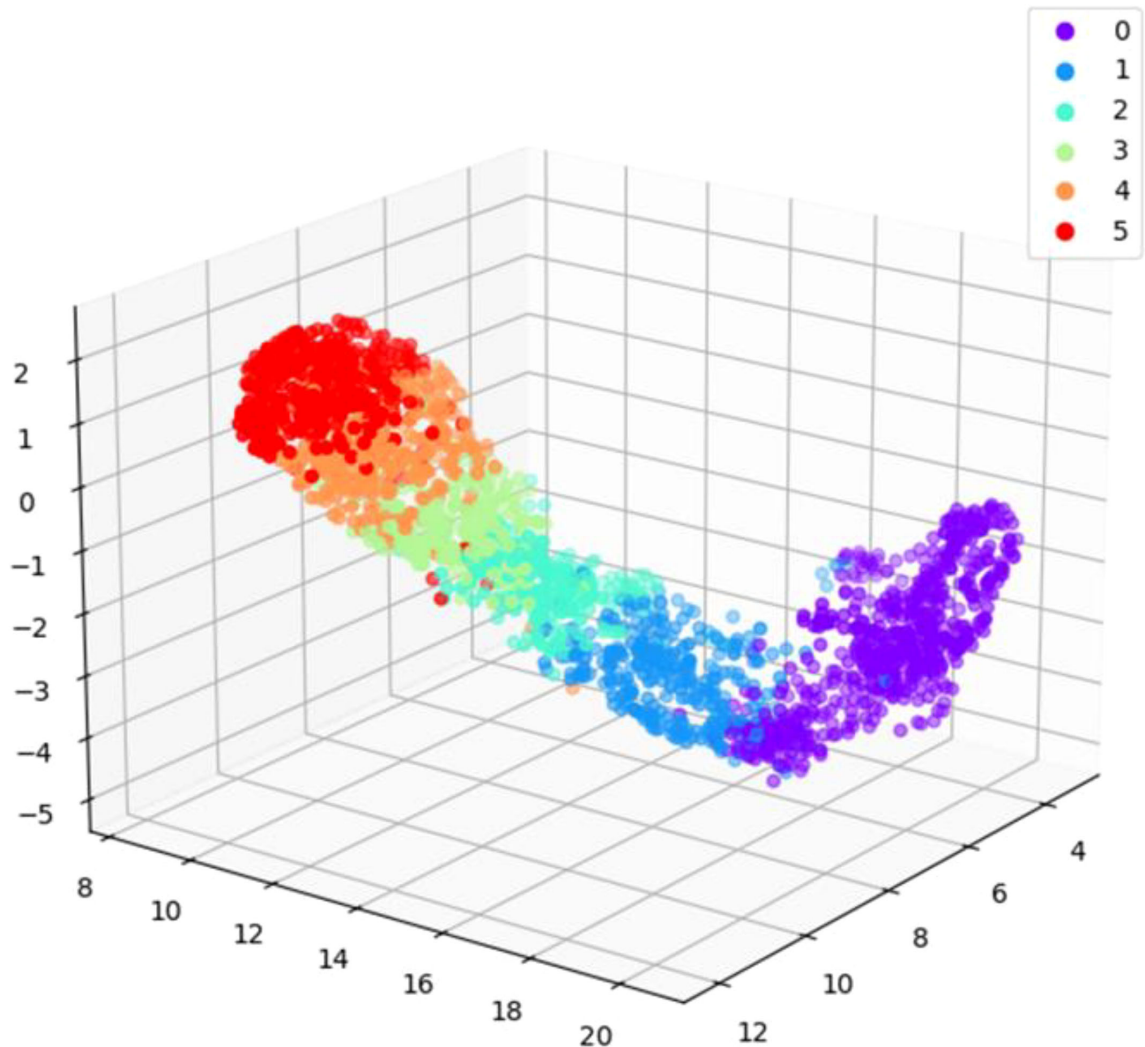
3D visualization of partial data sets on UMAP. 0 to 5 indicates increasing ripeness.

After resizing the strawberry patches to a size of 30×40 pixels, the pixel values of each patch were inputted into UMAP as the original high-dimensional data for 1000 iterations. The algorithm was implemented by umap of the python third-party tool library. The size of local neighborhood and effective minimum distance were respectively set to 25 and 0.4 for iteration. By reducing the initial data to three-dimensional space through the UMAP algorithm, we can observe the distribution of strawberries with different ripeness levels (**Figure 2**). Strawberries at different ripeness levels exhibit distinct boundaries and tend to cluster together based on their ripeness. This observation confirms the correctness of strawberry image annotation to a certain extent. But some points have large deviations, and we checked the strawberry patch annotations corresponding to these points. Then based on this result, the annotations of some images in the dataset were modified to improve the quality of manual annotations, making them more suitable for subsequent training tasks.

### 2.3 The overall processing flow of strawberry image

**Figure 3:**
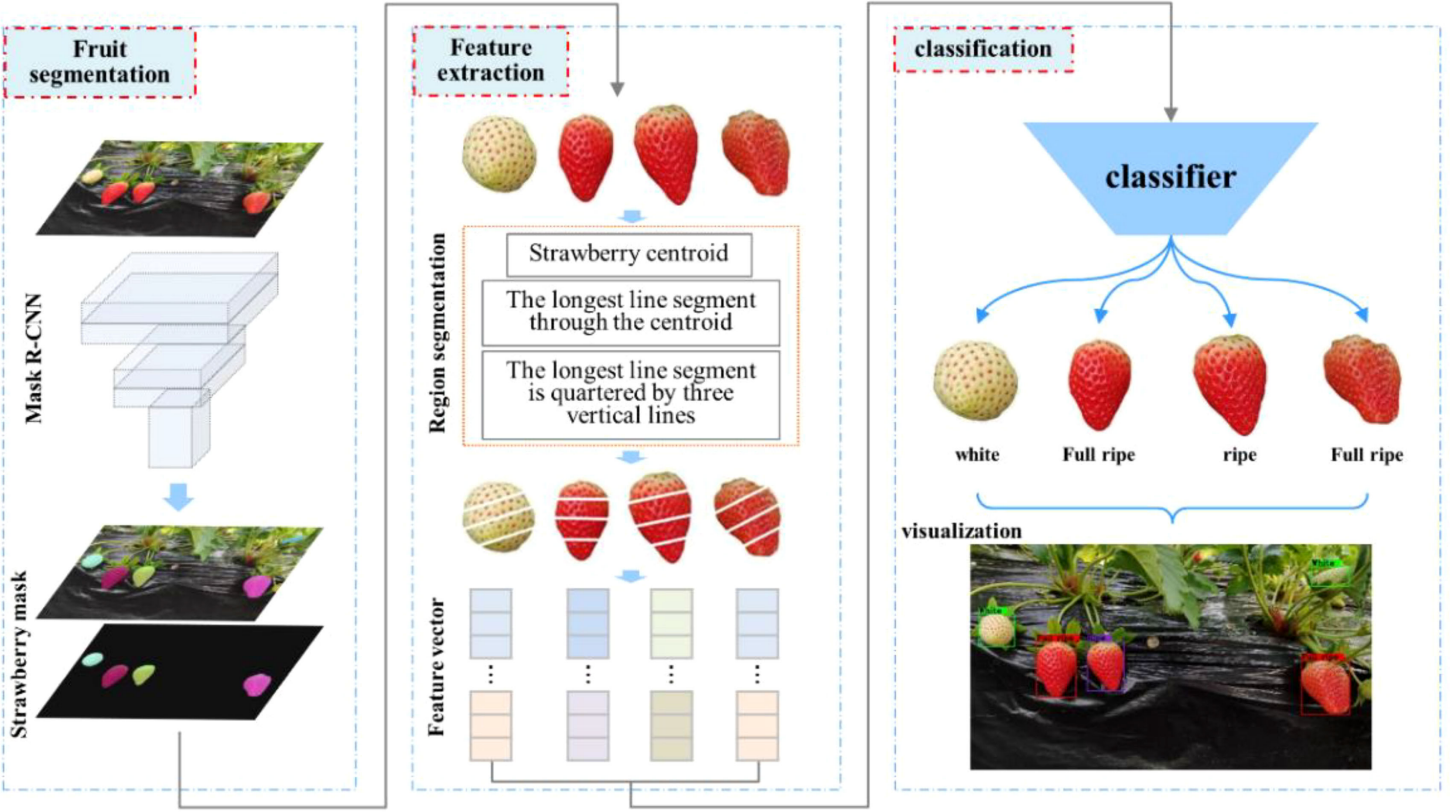
Flow chart of strawberry image processing.

The image processing flow is shown in **Figure 3**. First, the initial image is input into the Mask R-CNN network for strawberry instance segmentation, which generates a mask map. Next, each strawberry instance is segmented using the corresponding mask and divided into four sub-regions to extract features. Finally, the extracted feature values are input into a classifier to determine the ripeness level, resulting in the final visualization on the initial image. The ripeness detection of strawberries can be completed through the above three steps.

### 2.4 Strawberry detection model

Convolutional neural networks have strong feature extraction capabilities. However, in common convolution operations, the convolution is typically performed using multiple sets of convolution kernels of the same size, and the individual channels are then summed to obtain feature maps. The common convolution operation mode is the same, resulting in a limited richness of the learned feature representation. Therefore, the final segmentation results may exhibit shortcomings such as unclear object edges and incomplete segmentation of large objects (Pérez-Borrero et al., 2020). However, the utilization of self-calibrated convolutions can to a certain extent mitigate the above target segmentation issues. The Mask R-CNN instance segmentation model with self-calibrated convolutions will be explained in detail below.

#### 2.4.1 Self-calibrated convolutions

**Figure 4:**
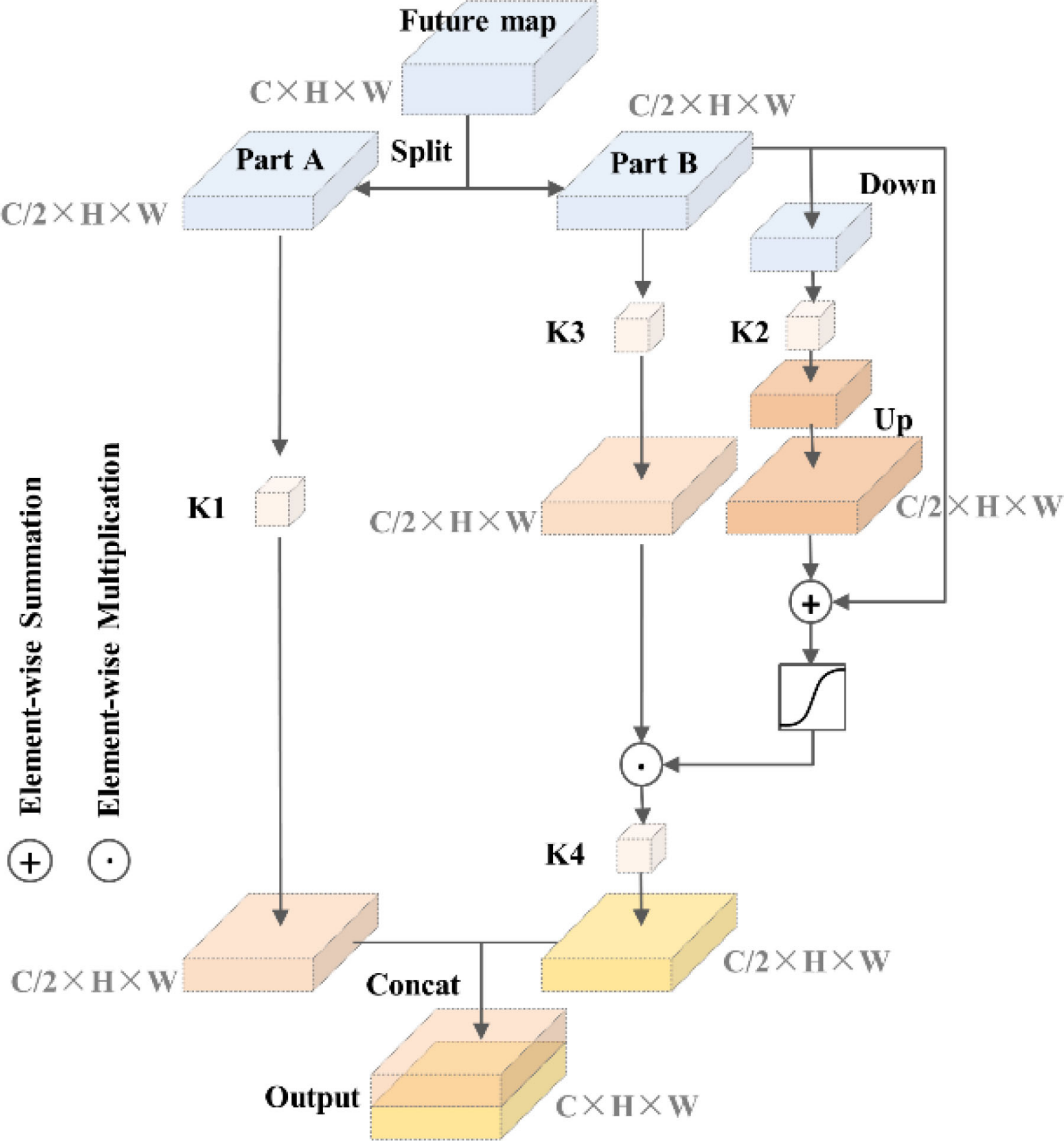
Self-calibrated convolutions structure.

A larger receptive field means that CNN can extract richer semantic information. In the traditional convolution process, the convolution kernels in same size result in fixed receptive fields, which may lack the capability to capture higher-level semantic information from a larger receptive field. The idea of self-calibrated convolution is to use deep features with a larger receptive field (such as strawberry advanced global information) to calibrate shallow features with richer position information (such as strawberry shape contour information) (Liu et al., 2020). The conventional convolutional layer applies a convolution operation to the feature map using a set of convolution kernels (K) of identical size. The self-calibrated convolution technique involves dividing the set of convolution kernels (K) into four parts, K1, K2, K3, and K4, and each part performs distinct convolution operations. Assuming that the number of input and output channels is the same, and the shape of K is (C, C, w, h), then the shape of K1 to K4 is (C/2, C/2, w, h). The details are shown in **Figure 4**. The input feature maps are divided into two parts, Part A and Part B. The K2 branch feature maps are first down-sampled to make it have a larger receptive field, and then convolution operation and up-sampling are performed with K2. Subsequently, the upsampling results are added to the feature maps of part B, and these results are then mapped to a weight value ranging from 0 to 1. This weight value assists in the convolution operation of the K3 branch, thereby achieving the goal of calibration. Finally, Part A and the calibrated Part B are concatenated after K1 and K4 convolution operations to obtain the final output feature maps.

The self-calibrated convolutions can effectively expand the receptive field and make the target positioning more complete and accurate without introducing additional parameters and complexity. The growth of strawberries in the field is influenced by a multitude of environmental factors, which often leads to variations in their sizes. The receptive field of common convolution is fixed and cannot adapt to changes in strawberry size. To address this limitation, the self-calibrated convolutions module is introduced to enhance the feature extraction results.

#### 2.4.2 Mask R-CNN combined with self- calibrated convolutions

**Figure 5:**
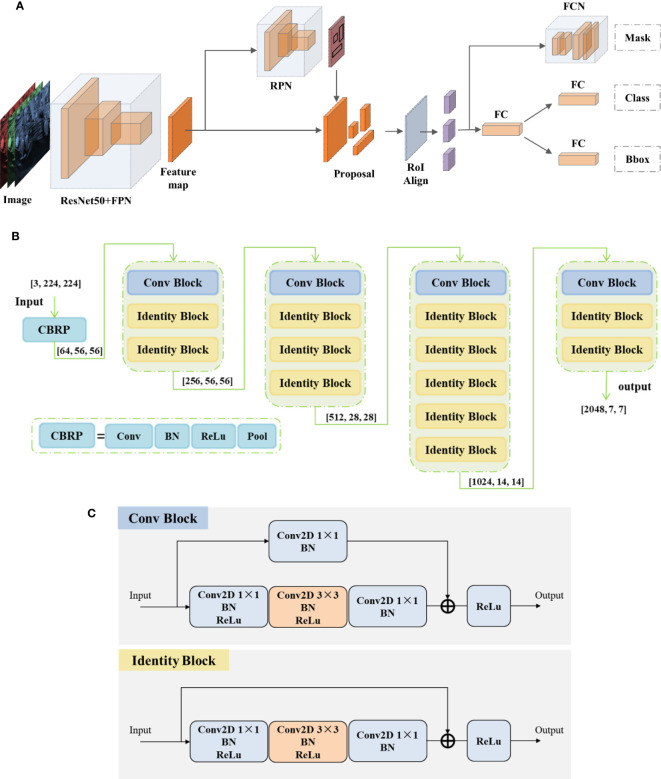
**(A)** Mask R-CNN network architecture. **(B)** ResNet50 network architecture. **(C)** Building block architecture.

Mask R-CNN (He et al., 2017) is a convolutional neural network designed for instance segmentation tasks, and it can segment fruits from complex natural environments (Ge et al., 2019; Yu et al., 2019; Huang et al., 2020). Mask R-CNN uses ResNet50/ResNet101 (He et al., 2016) as the backbone network and FPN (Lin et al., 2017) as the neck. Its head is the Faster R-CNN (Ren et al., 2017) head and adds a Mask head branch for pixel-level image segmentation. In order to reduce the computational cost, ResNet50 is selected as the backbone network. The Mask R-CNN network structure is shown in **Figure 5A**.

To enhance the performance of Mask R-CNN and achieve more accurate strawberry segmentation, the aforementioned self-calibrated convolutions are integrated into the original network. ResNet50 is constructed by stacking multiple building blocks, which consist of convolutional blocks and identity blocks. The architectural details of ResNet50 can be found in **Figure 5B**. It is worth mentioning that in **Figure 5B**, the last average pooling layer and fully connected layer of the original ResNet50 architecture are omitted. Convolutional block has a structure similar to identity block, which consists of a series of 1 × 1 convolution and 3 × 3 convolution, but the former has one more 1 × 1 convolution calculation in upper branch, as shown in **Figure 5C**. The self-calibrated convolution module can improve the network feature extraction results, so the convolution calculation of 3 × 3 convolution layers in all building blocks are replaced by self-calibrated convolutions.

#### 2.4.3 Model training

The training of DL model performed under the environment of Intel(R) Core(TM) i7-10700KF CPU @ 3.80GHz, 10 GB NVIDIA GeForce RTX 3080 GPU and 32 GB of RAM. The network was built through MMDetction open source tool library on the basis of PyTorch DL framework. In the training process, the horizontal flip data augmentation was performed randomly to prevent overfitting. The SGD optimizer was used for back-propagation to update the network parameters. The learning rate decay strategy was applied in the model training, and the learning rate was multiplied by 0.1 at the 15th, 20th, and 25th epoch to gradually reduce the learning rate. The model had been converging when the epoch was set as 30, so we saved the training results of each epoch and selected the best one on the validation set as test model. The specific hyperparameters are shown in **Table 2**.

**Table 2:** Hyperparameters of model training.

Hyperparameter	Value
Learning Rate	0.02
Momentum	0.9
Optimizer	SGD
Batch Size	3
Epoch	30
Wormup Iterations	500
Decay Steps(epoch)	[15,20,25]

### 2.5 Feature extraction method

**Figure 6:**
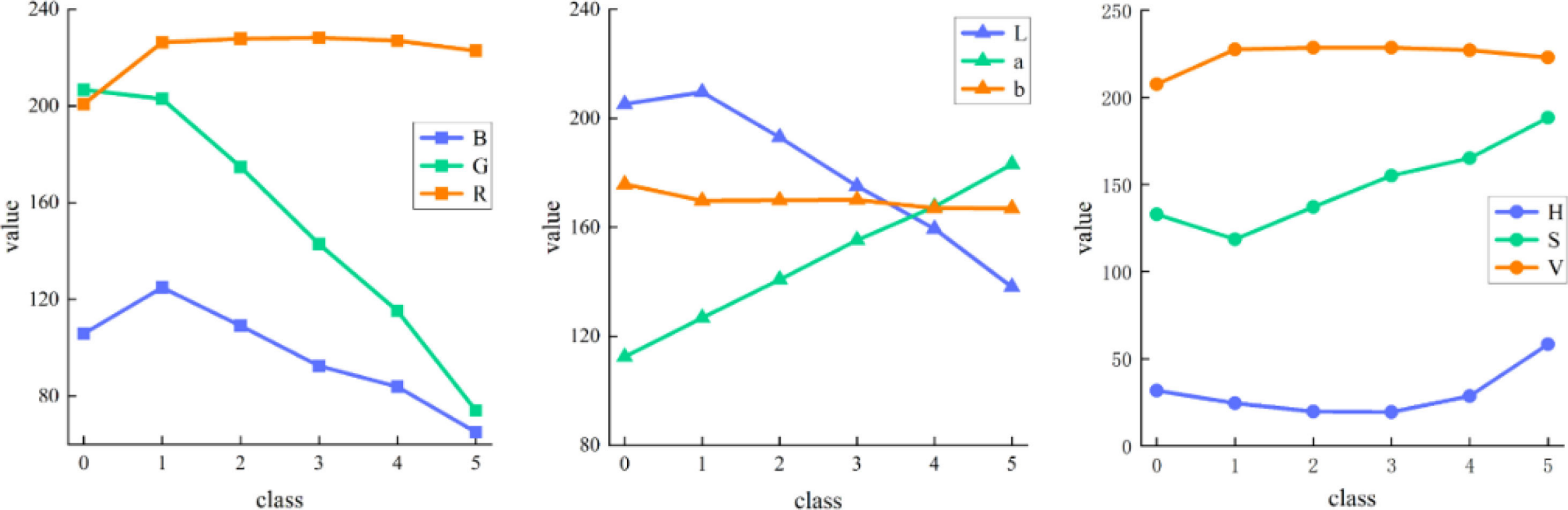
Mean values of different color spaces. 0 to 5 indicates increasing ripeness.

First, the RGB images were converted to HSV and Lab color spaces, and the color features of strawberry patches were counted. Then the change relationship between the color mean of each channel and the ripeness level can be observed in **Figure 6**. The ordinate in the figure represents the mean value of strawberry foreground pixels, and the abscissa from 0 to 5 represents the gradually increasing ripeness. It can be seen from the figure that the average color values of channels B, G, and L show an obvious decreasing trend with the increase of strawberry ripeness. The mean color values of channels a and S increased significantly with the increase of ripeness. There is a certain correlation between the color feature value of strawberry and its ripeness, among which the channel a is the strongest, but the channels R, b, H, and v are not obvious enough. Channels B, G, L, a, and S are selected for strawberry color feature extraction based on region segmentation to reduce computational complexity and eliminate noise interference in other data.

**Figure 7:**
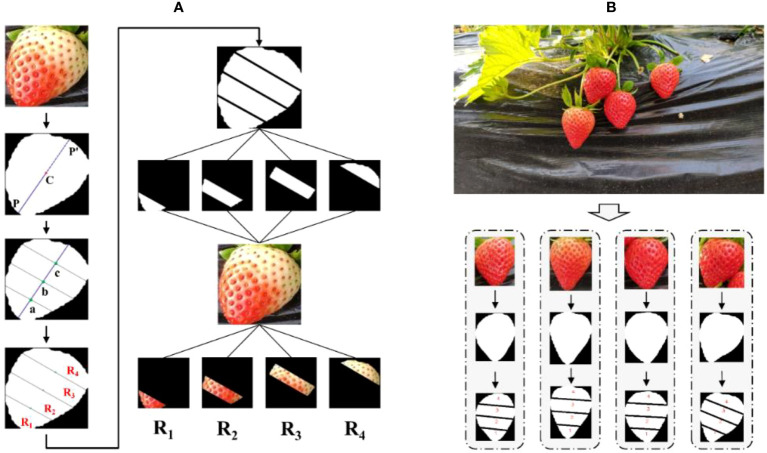
**(A)** Flow chart of strawberry region segmentation. **(B)** Example of strawberry region segmentation results.

To extract strawberry features effectively, the strawberry is divided into four sub-regions, and the color mean of each region is extracted as the color feature of the strawberry. Before feature extraction, it is necessary to divide and mark the strawberry, which can be accomplished through the following steps. The specific process is shown in **Figure 7A**.

Step 1: Determine the strawberry centroid. After processing the original image with Mask R-CNN, a masked binary image of strawberry will be generated. The mask coordinate $\left(x_{i},y_{i}\right)$ and Eq. (7) are used to determine the center of mass coordinate *C* $\left(x_{0},y_{0}\right)$ of strawberry.


(7) $$\{\begin{array}{c} x_{0}=\frac{\sum_{i=1}^{N}p_{i}x_{i}}{\sum_{i=1}^{N}p_{i}}\\ y_{0}=\frac{\sum_{i=1}^{N}p_{i}y_{i}}{\sum_{i=1}^{N}p_{i}} \end{array}$$

where *N* is the total number of strawberry pixels, and *p*_i_ is the value of the *i*-th pixel.

Step 2: Find the longest line segment through the centroid. The outer contour point *P*_i_ of the strawberry binary image can be expressed as $\left\{\left(x_{i},y_{i}\right)|1\leq i\leq M\right\}$ , and by traversing each outer contour point, *M* straight lines passing through the centroid *C* $\left(x_{0},y_{0}\right)$ can be obtained, which can be expressed as $\left\{\left(x,y\right)|A_{i}y+B_{i}x+C_{i}=0,1\leq i\leq M\right\}$ . These lines are traversed, and the distance from each contour point to the line is obtained using Eq. (8). Find the contour point *P*_i_’ at the minimum distance and use it as another approximate intersection of this line with the contour. When the minimum distance is 0, it indicates that the point is on the line (excluding the contour points that construct the line). This results in a total of *M* approximate intersections. Finally, each line has two intersections with the strawberry outline. The farthest set of intersection points are connected and used as the longest line segment *PP*’ through the strawberry’s centroid.


(8) $$d=\frac{\left|A_{i}x_{j}+B_{i}y_{j}+C_{i}\right|}{\sqrt{A_{i}^{2}+B_{i}^{2}}},\left(1\leq i\leq M,1\leq j\leq M\right)$$

Step 3: Find three vertical lines to divide the longest line segment into four equal parts. We can easily find the three coordinate points a, b, c on the line segment *PP*’ such that *PP*’ is divided into four equal parts. Then through these three points, three vertical lines *l*_a_, *l*_b_, *l*_c_ perpendicular to the line segment *PP*’ are obtained. Each vertical line approximately intersects with the strawberry contour at two points, which can be obtained by calculating the approximate intersection point in step 2.

Step 4: Area marking. The three sets of intersection points in step 3 are connected respectively, and the strawberry is divided into four sub-regions. The centroid coordinate *C* of each sub-region is calculated separately by Eq. (7). The sub-regions are sorted from bottom to top according to the value of *y*_0_ and marked as R_1_, R_2_, R_3_, R_4_. The purpose of region marking is to enable subsequent feature extraction in this order.

**Figure 7B** shows some examples of results after the strawberry region is automatically divided. It can be seen that each sub-region of strawberry is well segmented by three line segments, and the four sub-regions are correctly marked in order.

### 2.6 Classification method

According to the extracted strawberry features, selecting a classifier that matches the data type can maximize the classification effect. Strawberry features are high-dimensional data and have nonlinear characteristics. To fully leverage the performance of the classifier and enhance the accuracy of ripeness classification, the SVM (Support Vector Machine) was considered first. SVM is a linear classifier suitable for processing high-dimensional data. Due to its advantages of fast training speed, high accuracy, and good robustness, SVM has gained extensive usage in the field of image classification (Tu et al., 2018; Dhakshina Kumar et al., 2020). For comparison, we tried other classic machine learning methods, including LR (Logistic Regression), KNN (K-Nearest Neighbors), RF (Random Forest), and finally obtained the best classifier by comparative analysis. We used 5-fold hierarchical cross-validation and grid search methods to optimize the parameters of these classifiers. The optimized parameters were used as the final parameters of the model (**Table 3**).

**Table 3:** The main parameters of the different classifiers.

Classifier	Param
LR	‘c’: 0.7, ‘solver’: ‘newton-cg, ‘penalty’: l2
KNN	‘n_neighbors’: 12
RF	‘max_depth’: 20, ‘n_estimators’: 35
SVM	‘C’: 10, ‘kernel’: ‘rbf’, ‘gamma’: 0.0005

* ‘c’: reciprocal of penalty term coefficient, ‘penalty’:penalty item, ‘solver’: optimization method, ‘n_neighbors’: number of neighbors, ‘max_depth’: decision tree maximum depth, ‘n_estimators’: number of decision trees, ‘C’: penalty coefficient, ‘kernel’: kernel function, ‘gamma’: gamma coefficient.

## 3 Results

### 3.1 Evaluation methods

For segmentation tasks, we will compare the segmentation effects of Mask R-CNN’s backbone network before and after adding self-calibrated convolutions. For the task of strawberry ripeness classification, we will evaluate the classification performance of different classifiers using various combinations of color channels. Subsequently, we will identify the optimal classifier based on the results. Then we will use the optimal classifier to evaluate the classification effect of different feature extraction methods to illustrate the superiority of our proposed feature extraction method. Finally, the proposed method will be compared with the common CNN.

The following is an introduction to the model evaluation indicators. AP, AP.50, AP.75 are used to evaluate the segmentation effect of the model. F1 and accuracy are used to evaluate the classification performance of the classifier. AP represents the mean of the average precision under 10 IoU thresholds from 0.50 to 0.95 with 0.05 intervals, which is the most important evaluation metric for MS COCO competition. AP.50 represents the average precision when IoU=0.50, and AP.75 represents the average precision when IoU=0.75. IoU is the intersection and union ratio of the mask area. The average precision is the area under the P-R curve, which can be obtained from Eq. (9). P(r) is the P-R curve obtained from precision and recall. TP represents the number of positive samples correctly predicted. TN represents the number of negative samples correctly predicted. FP represents the number of positive samples that were incorrectly predicted. FN represents the number of negative samples that are incorrectly predicted.


(9) $$\{\begin{array}{c} \text{Precision}=\frac{TP}{TP+FP}\\ \text{Recall}=\frac{TP}{TP+FN}\\ \text{Average Precision}=\int^{\;}P\left(r\right)dr \end{array}$$

(10) $$\{\begin{array}{c} F1\;Score=\frac{2\times Precision\times Recall}{Precision+Recall}\\ Accuracy=\frac{TP+TN}{TP+TN+FP+FN} \end{array}$$

### 3.2 Detection performance of instance segmentation model

**Figure 8:**
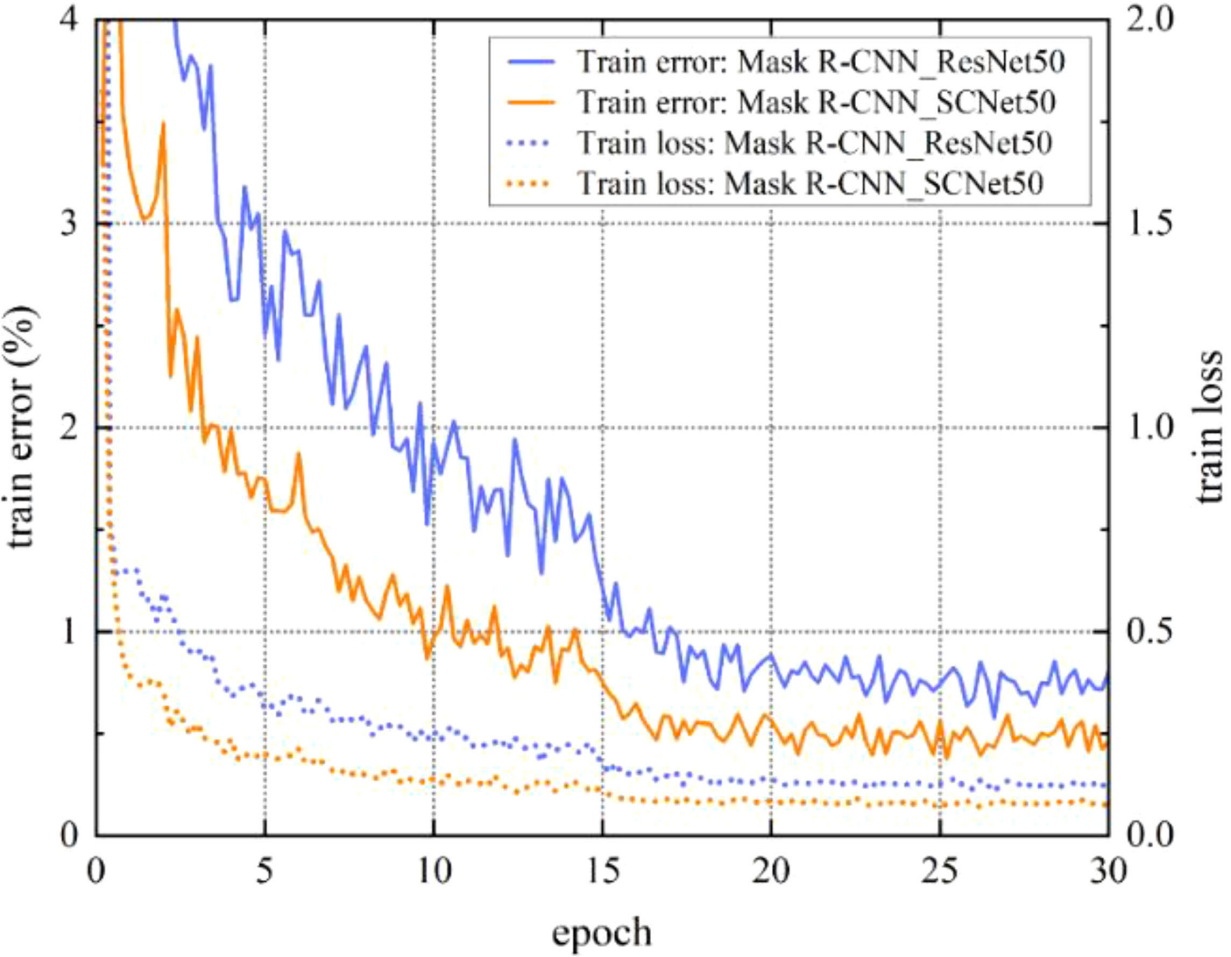
Model training loss and training error. SCNet50 is the backbone network with self-calibrated convolutions.

To assess the impact of the Mask R-CNN model improvement, we conduct a comprehensive comparison by considering the training phase, testing phase, and the final strawberry segmentation results. This allows us to observe the effectiveness of the model before and after the proposed enhancements. The loss curve and training error curve of the model are shown in **Figure 8**. It can be seen from the figure that the loss of the model begins to stabilize around 25 epochs, and the model has converged at 30 epochs. After incorporating self-calibrated convolutions to the original ResNet50 backbone network, the model exhibits lower loss during convergence, indicating an improved fit of the model. Additionally, it is evident that the training error of SCNet50, after incorporating self-calibrated convolutions, is lower than that of ResNet50. This demonstrates that the inclusion of self-calibrated convolutions leads to an improvement in model accuracy to a certain extent.

During the training process, the best performing model on the validation set was saved. Then the final performance of the model was verified on the test set. The test results of the model are shown in **Table 4**. Mask R-CNN utilizing SCNet50 as the backbone network exhibits a higher average precision compared to using ResNet50. The AP of SCNet50 reaches 0.937, which is 0.039 higher than that of ResNet50, and the AP.50, AP.75 are also improved by 0.021 and 0.032, respectively. But in inference speed, the FPS of SCNet50 is reduced, which is within our allowable range. The feature extraction ability of ResNet50 is improved after adding self-calibrated convolutions. Not only did the model perform better on training, it also performed well on testing. This indicates its strong generalization ability, but at the same time it also increases a certain time cost.

**Table 4:** The test results of instance segmentation model.

Model	Backbone	AP	AP.50	AP.75	FPS
Mask R-CNN	ResNet50	0.898	0.958	0.937	19.4
	SCNet50	0.937	0.979	0.969	18.2

SCNet50 is the backbone network with self-calibrated convolutions.

**Figure 9:**
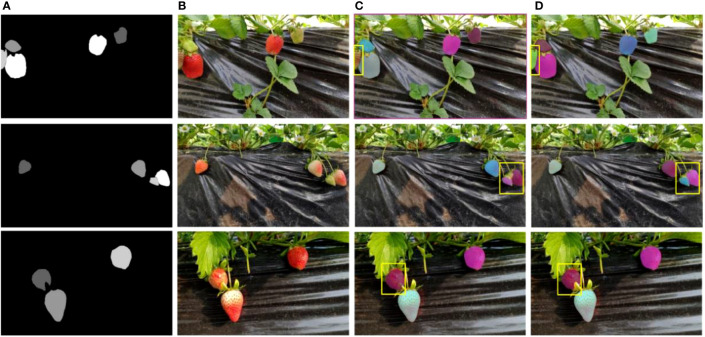
Strawberry segmentation results. The yellow rectangles indicate the area to be compared. **(A)** Ground truth. **(B)** Initial images. **(C)** ResNet50 results. **(D)** SCNet50 results.

The final segmentation results of strawberry are shown in **Figure 9**. The strawberry marked by the yellow box in the first row of picture has missed detection. The reason may be that the surrounding background color is similar to the strawberry. The strawberry in the picture on the right is successfully detected because SCNet50 extracts richer semantic information. It is still capable of identifying the target even in cases where the background and the target have similar colors. In the second row of the figure, the overlapping strawberries marked by the yellow box on the left are not completely segmented. In the third row of the picture, the strawberry marked by the yellow box on the left is incorrectly identified as part of the strawberry because the strawberry is occluded by the leaf. These erroneous segmentations will have an impact on subsequent strawberry ripeness classifications. From **Figure 9D**, it can be observed that the aforementioned erroneous segmentations have been effectively improved, and overall, the edges of the strawberries are more detailed. By adding self-calibrated convolutions, the model has a larger receptive field and can generate richer feature representations, making target positioning more accurate.

**Figure 10:**
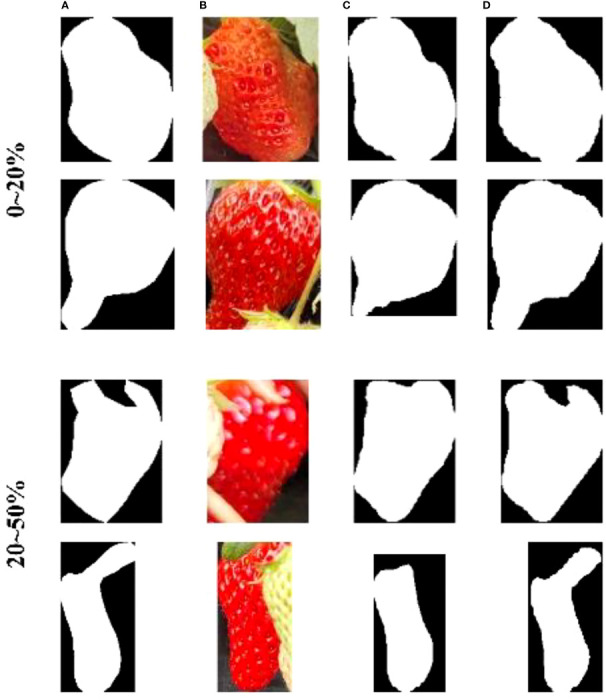
Segmentation examples under different occlusion areas. **(A)** Ground truth. **(B)** occlusion strawberries. **(C)** ResNet50 results. **(D)** SCNet50 results.

To further analyze the model’s robustness against occlusion, we have compared the strawberry segmentation accuracy under different occlusion areas (**Table 5**). We manually counted the number of strawberries covered by stalks, leaves, and other strawberries in the test set, dividing them into two categories: 0-20% and 20-50% based on occlusion area. As shown in **Table 5**, SCNet50 demonstrates higher accuracy in segmenting strawberry when faced with occlusion interference, particularly under the 20-50% occlusion area where its mean IoU improves by 0.056 compared to ResNet50. Examples of the segmentation results can be found in **Figure 10**.

**Table 5:** Mean IoU comparison of models under different occlusion areas of strawberries.

Model	Backbone	0~20%	20~50%
Mask R-CNN	ResNet50	0.896	0.849
	SCNet50	0.918	0.905

### 3.3 Strawberry color feature extraction

**Figure 11:**
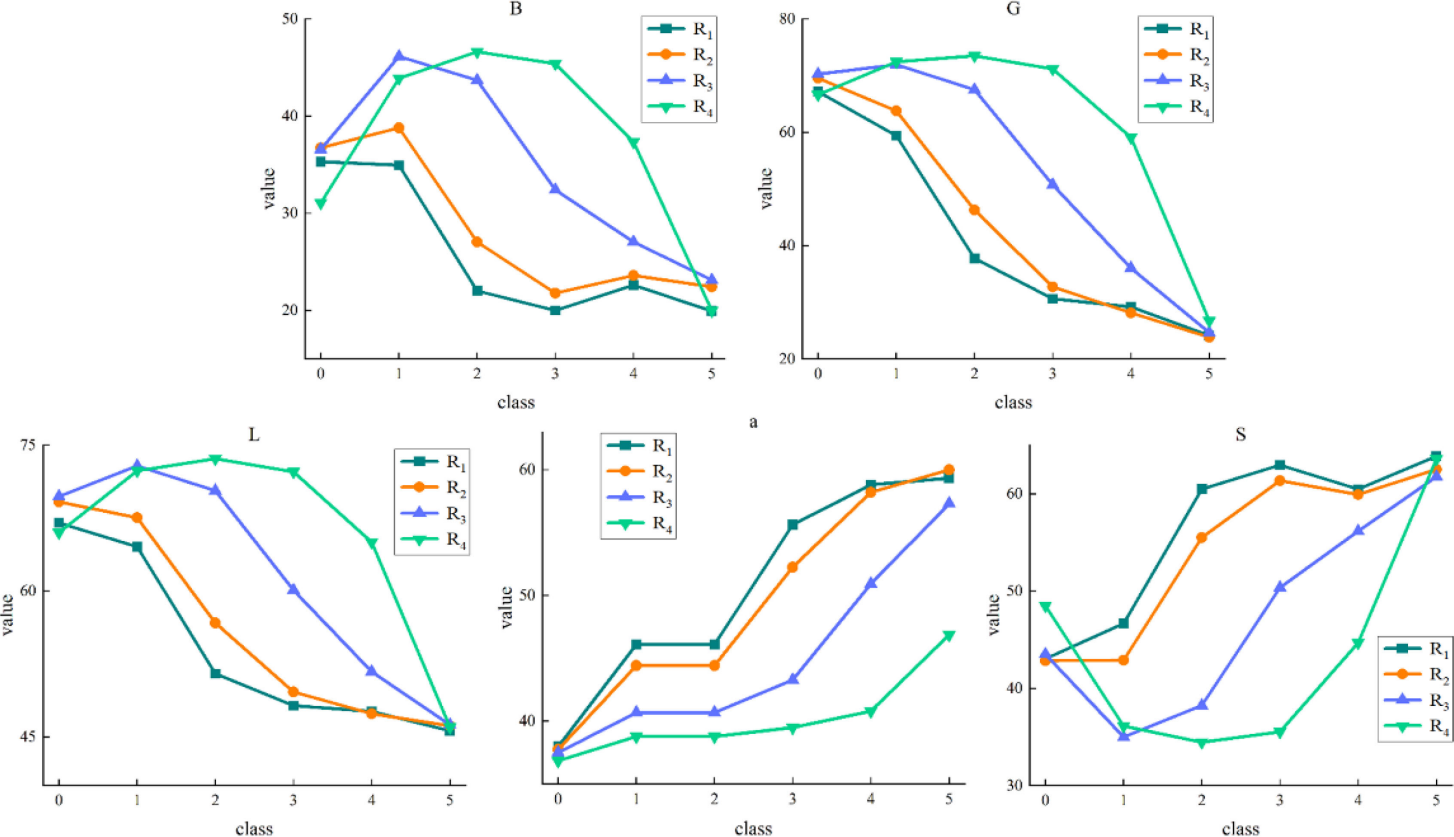
Variation trend of color feature values in strawberry sub-regions. 0 to 5 indicates increasing ripeness.

We employ the approach outlined in Section 2.5 to extract the color features of strawberries. By calculating the color mean of each sub-region in each channel, we can observe the trends and variations in these color features. The results are shown in **Figure 11**. The ordinate in the figure represents the average pixel value of the strawberry sub-region, and the abscissa from 0 to 5 represents the gradually increasing ripeness. With the change of sub-regions R_1_ to R_4_, the color feature values in channels B, G, L show an increasing trend at the same ripeness stage, and show a decreasing trend in channels a and S. In addition, the color feature values of the B, G, and L channels have similar trends with ripeness. Among them, R1, R2, and R3 decrease with increasing ripeness, while R4 gradually increases in the first three ripeness stages and then gradually decreases in the last three ripeness stages. Channel a and S have a gradual rise in overall. Among them, R_4_ gradually decreases in the first three ripeness stages in the channel S, and the latter three ripeness stages gradually increases. As the strawberry ripeness increases, we observe a systematic change in the color feature values of the different sub-regions across each channel. This consistent pattern proves beneficial for the effective functioning of subsequent classifiers.

### 3.4 Classification of strawberry ripeness

The classification results of strawberry ripeness are shown in **Table 6**. From the perspective of each color channel, Channel a achieves the highest classification accuracy when considered individually. Among the classifiers, SVM shows the best performance with an accuracy of 0.850. It can be easily explained from **Figure 11**. The color feature values of Channel a increase with the ripeness, indicating a strongest correlation and providing favorable conditions for classifier judgment. In the combined channels, as the number of channels increases, the accuracy of the LR and SVM classifiers gradually increase. However, in the KNN classifier, BGa, GaS, BGaS, and BGLaS under the combination channels have decreased accuracy compared to Ga. This shows that the features of the B, S, and L channel have a certain interference effect on the classification effect of KNN. In the RF classifier, the results of GaS have decreased compared to Ga, and the results of BGLaS have decreased compared to BGaS. This indicates that the feature information from the S and L channels is redundant for the classifier, and including this data dose not lead to an improvement in performance. When all channels are combined, SVM achieves the highest classification accuracy of 0.866, demonstrating its effectiveness in handling high-dimensional data. The classification performance of RF is second only to SVM, with an accuracy of 0.861 achieved using the BGaS channel. The inaccurate classification may be due to abnormal distribution of surface color in some strawberries or the strawberries not being in a downward fruit-hanging posture overall. These will cause outliers in feature extraction, which will lead to wrong classification.

**Table 6:** Classification accuracy of different color channels.

	B	G	L	a	S	Ga	BGa	GaS	BGaS	BGLaS
LR	0.651	0.768	0.693	0.842	0.704	0.840	0.850	0.849	0.854	0.857
KNN	0.645	0.783	0.724	0.844	0.696	0.839	0.828	0.829	0.823	0.819
RF	0.622	0.791	0.705	0.846	0.659	0.860	0.860	0.856	0.861	0.849
SVM	0.639	0.770	0.710	**0.850**	0.697	0.854	0.863	0.859	0.863	**0.866**

Values in bold mean the highest classification accuracy under single channel and combined channel among all classifiers.

**Figure 12:**
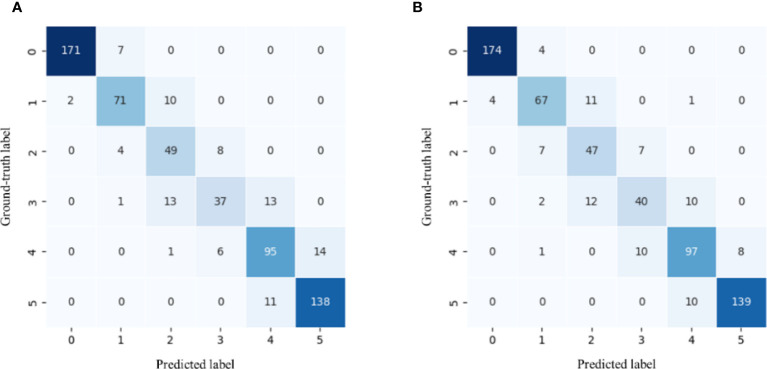
**(A)** RF confusion matrix. **(B)** SVM confusion matrix. 0 to 5 indicates increasing ripeness.

**Figure 12**. is the confusion matrix when RF and SVM respectively obtain the best results. Except in Breaking (label 1) and Turning-1 (label 2), SVM is better than RF. According to the above analysis, SVM is selected as the suitable classifier.

**Figure 13:**
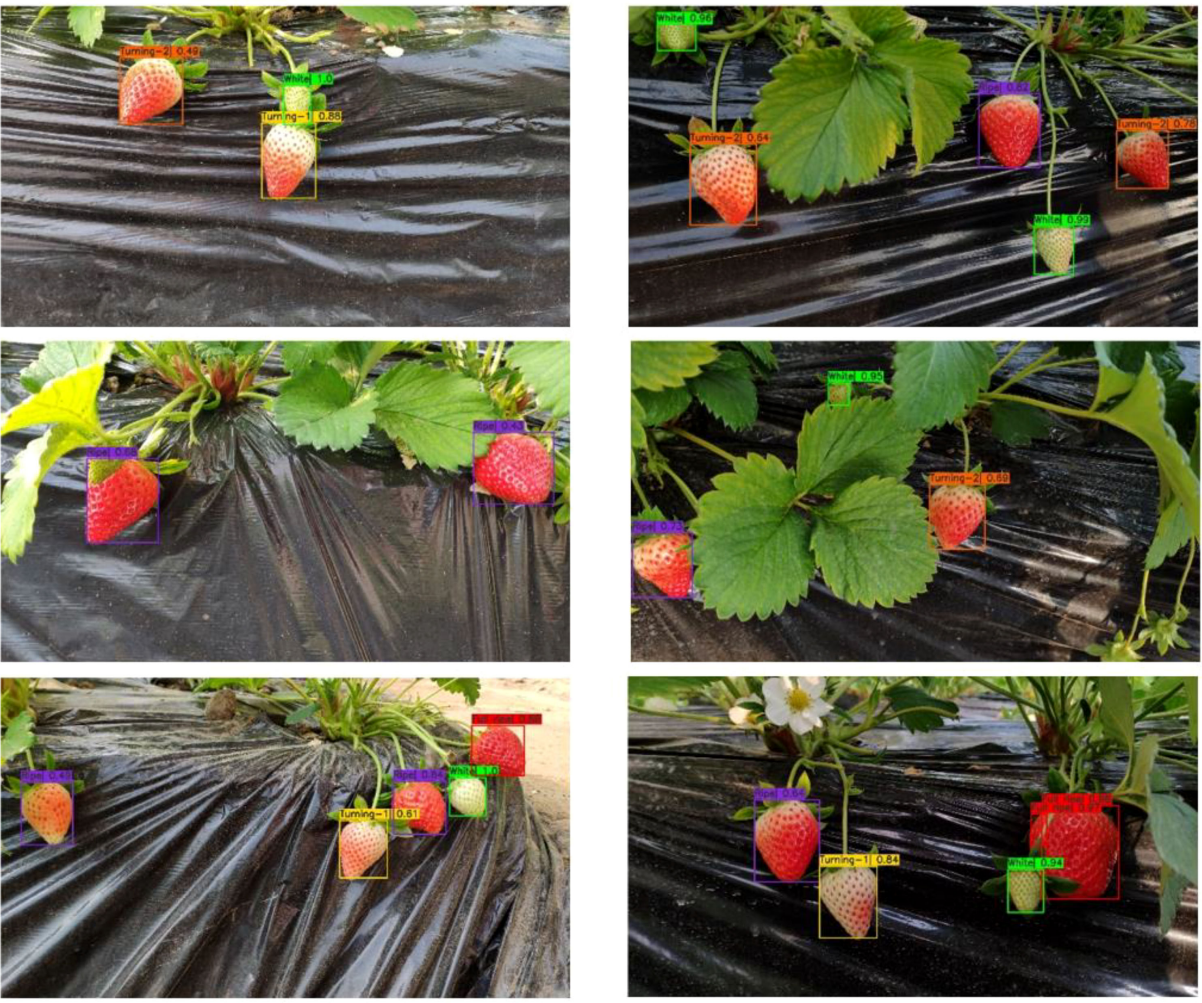
The visualization results of strawberry ripeness detection.

The final detection results of strawberry ripeness is visualized (**Figure 13**). It is worth mentioning that the probabilities in the results represent SVM classification probabilities. It is important to mention that in the left image of the second row, there was an undetected green strawberry. This is because it is not considered in the model training and does not belong to any of the six ripeness categories. Strawberries can be detected in both frontlighting and backlighting environments, as shown in the first row of images. Even under slight occlusions, as depicted in the second row, the strawberry ripeness level can still be successfully identified. However, in the right image of the first row, the strawberry is severely occluded, and the instance segmentation model failed to detect the strawberry, resulting in the inability to recognize its ripeness subsequently. In the last image, the same strawberry was detected twice, resulting in duplicate detections. This is because the strawberry is occluded by the stalk, and the instance segmentation model mistakenly recognizes it as two instances, causing subsequent tasks to treat it as two objects for processing. In general, the overall performance of the model is largely affected by the segmentation performance. When the first-stage segmentation model failed to detect or misdetected objects, the model was unable to predict strawberry ripeness, so the predictions could not be reversed.

### 3.5 The effect of different sub-regions on classification results

**Table 7** is the classification results of strawberry ripeness under the SVM classifier based on the color features of different sub-regions. In terms of the single sub-regions’ effects, except for the B channel, R_3_ consistently exhibits the highest classification accuracy. In terms of the combination effects of sub-regions, as the number of sub-regions increases, the feature information is more diverse and comprehensive. Consequently, this leads to enhanced classification accuracy for each single channel. In order to further analyze the specific contributions of each sub-region to different ripening stages of strawberries, we extracted the color feature values under the combined channel BGLaS. Subsequently, we utilized the SVM classifier to classify the ripeness. The number of correct classification labels was counted, as shown in **Table 8**. First of all, the sub-region with the highest classification accuracy is R_3_, which is 68.15%. This is consistent with the result that R_3_ in **Table 7** basically maintains the highest accuracy in a single channel. In the White stage, the accuracy of R_2_ demonstrates the highest performance, while in the Breaking and Turning-1 stages, the accuracy of R_1_ exhibits the highest level of accuracy. The classification effect of Turning-2 mainly depends on R_3_, which contributes the most to the classification effect of this stage. Ripe and Full ripe both bring the most obvious classification effect under R_4_.

**Table 7:** Classification results of different sub-regions under single channel.

	R_1_	R_2_	R_3_	R_4_	R_3_R_4_	R_1_R_3_R_4_	R_2_R_3_R_4_	R_1_R_2_R_3_R_4_
B	0.488	0.493	0.481	0.487	0.588	0.630	0.625	0.639
G	0.601	0.604	0.621	0.593	0.694	0.766	0.768	0.770
L	0.524	0.539	0.553	0.551	0.639	0.682	0.699	0.710
a	0.590	0.642	0.710	0.690	0.776	0.846	0.840	0.850
S	0.510	0.521	0.522	0.502	0.625	0.693	0.671	0.697

**Table 8:** Contribution of different sub-regions to each ripeness stage.

Class (number)	R_1_	R_2_	R_3_	R_4_
White (178)	172(96.63%)	174**(97.75%)**	169(94.94%)	164(92.13%)
Breaking (83)	62**(74.70%)**	61(73.49%)	51(61.44%)	46(55.42%)
Turning-1 (61)	41**(67.21%)**	36(59.02%)	26(42.62%)	1(1.64%)
Turning-2 (64)	1(1.56%)	21(32.81%)	37**(57.81%)**	26(40.63%)
Ripe (116)	28(24.14%)	43(37.07%)	74(63.79%)	89**(76.72%)**
Full ripe (149)	124(83.22%)	127(85.23%)	124(83.22%)	136**(91.28%)**
Total (672)	428(63.69%)	437(65.03%)	485(68.15%)	433(64.43%)

Values in bold mean the highest classification accuracy in each ripeness stage.

**Figure 14:**
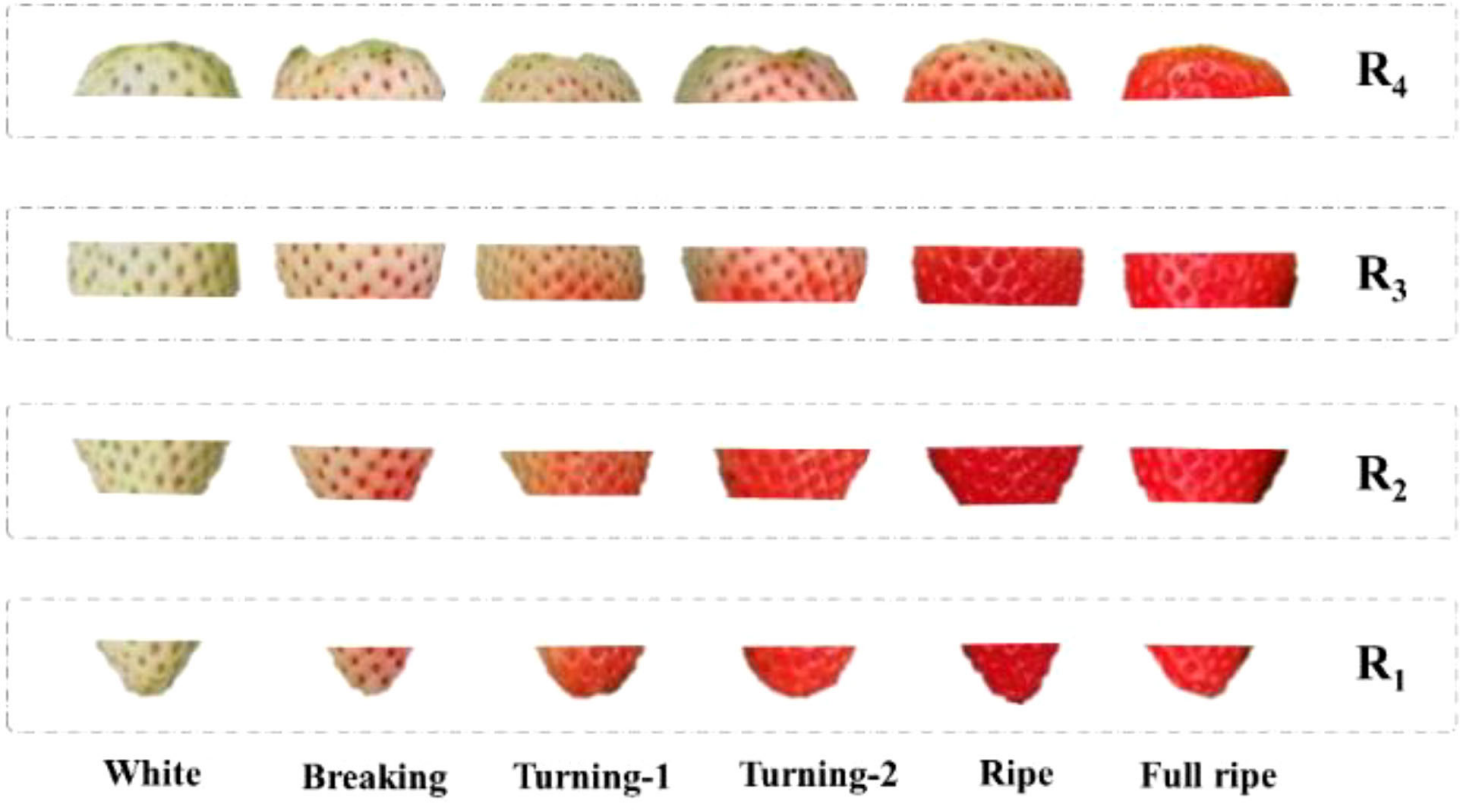
Examples of sub-regions at different ripeness levels.

The increase of strawberry ripeness is basically accompanied by the continuous expansion of the surface red area from bottom to top, as shown in **Figure 14**. During the early stages of strawberry ripeness, the red area is small. The color change primarily occurs in the lower half of the strawberry, while the color of the upper half remains relatively unchanged. Therefore, the color differences of White, Breaking and Turning-1 in the sub-regions R_1_ and R_2_ are relatively large, which is conducive to the judgment of the three ripeness levels. In the later stages of strawberry ripening, the lower half of the strawberry basically turns red, and the green area of the upper half gradually diminishes. This color difference is also helpful in judging the ripeness of Turning-2, Ripe and Full ripe. Therefore, when considering **Table 8**, it becomes evident that R_1_ and R_2_ play a significant role in determining the first three ripeness levels. On the other hand, R_3_ and R_4_ exhibit greater influence in discerning the last three ripeness levels. In **Table 8**, the accuracy of each sub-region of the White stage is higher, because the whole surface of the strawberry in the White stage is light green. No matter under which sub-region, its color value is obviously different from other stages.

### 3.6 Comparison of different classification methods

To validate the superiority of the proposed feature extraction method, we compared it with the common manual feature extraction methods. Typical manual feature extraction methods can be divided into two categories: 1) taking each pixel as a feature value; 2) taking the pixel mean of the foreground target as a feature value. **Table 9** shows the classification results of different strawberry color feature extraction method. Method 1 is to resize the strawberry block cropped by the rectangular frame to 30×40, while method 2 is to take the mean value of the segmented strawberry foreground pixels as the feature value. **Table 9** clearly demonstrates that the accuracy of the proposed method is higher than other methods across all channels. The highest accuracies of method 1 and method 2 are 0.811 and 0.826, which are 0.055 and 0.040 lower than the proposed method respectively. Method 1 primarily emphasizes full-image pixel classification, placing excessive emphasis on pixel position information. This approach may result in inaccurate classification, particularly when dealing with horizontally arranged strawberries that undergo deformation during the resizing process. Method 2 primarily emphasizes foreground pixel classification and relies on the color mean value as a classification feature. However, it overlooks pixel position information, which ultimately results in inaccurate classification. While the color feature extraction based on region segmentation in the proposed method takes into account both the positional information of the red region as it changes with ripeness and the pixel-level information. Therefore, the proposed method can obtain more informative features for strawberry ripeness classification.

**Table 9:** SVM classification accuracy of different feature extraction methods.

	B	G	L	a	S	Ga	BGa	GaS	BGaS	BGLaS
Method 1	0.612	0.745	0.676	0.762	0.676	**0.811**	0.806	0.800	0.799	0.796
Method 2	0.520	0.692	0.614	0.786	0.561	0.800	0.812	0.821	0.821	**0.826**
Proposed	0.639	0.770	0.710	0.850	0.697	0.854	0.863	0.859	0.863	**0.866**

Values in bold mean the highest classification accuracy for each method.

The fruit ripeness classification based on CNN is also a widely adopted method. Therefore, we conducted a comparison between the proposed method and commonly used CNN models. The parameter settings of CNN model training are consistent. The learning rate and batch size are 0.001 and 16, respectively. The model uses the SGD optimizer and iterates for 30 epochs to train the parameters. The learning optimization strategy adopts the MultiStepLR method, and the learning rate decays at the 18th, 24th, and 27th epoch respectively. Gaussian blur and horizontal flip data augmentation are randomly performed on the image during training. The experimental results are shown in **Table 10**. Except that the F1 score of the proposed method is lower than AlexNet and ResNet18 in the Turning-1 and Turning-2 stages, the rest of the ripeness stages show better classification results. The classification error rate of the proposed method is primarily concentrated in the Turning-1 and Turning-2 stages, because there are more strawberries in transitional ripeness stages between Turning-1 and Turning-2 stages. Their features are very similar, which can easily result in the classification results to swing between these two stages.

**Table 10:** Test results of different classification methods.

Ripeness category	AlexNet				ResNet18				Proposed			
	P	R	F1	Acc	P	R	F1	Acc	P	R	F1	Acc
White	0.94	0.99	0.96	0.848	0.99	0.93	0.96	0.856	0.98	0.98	0.98	0.866
Breaking	0.86	0.75	0.80		0.73	0.94	0.73		0.83	0.81	0.82	
Turning-1	0.69	0.74	0.71		0.79	0.69	0.79		0.67	0.77	0.72	
Turning-2	0.69	0.72	0.72		0.72	0.61	0.72		0.70	0.62	0.66	
Ripe	0.77	0.81	0.79		0.78	0.81	0.78		0.82	0.84	0.83	
Full ripe	0.93	0.87	0.90		0.92	0.93	0.93		0.95	0.93	0.94	

Acc means accuracy.

## 4 Discussion

In this study, we have developed a method that combines Mask R-CNN and region segmentation to accurately assess the ripeness of strawberries in the field. The method proposed in this paper is compared with existing research work (**Table 11**). In most cases, managing strawberry planting, including monitoring fruit growth status and predicting fruit yield, needs to be done in a natural environment rather than indoors. In earlier studies, the majority of research was conducted within the confines of an indoor setting. This highly structured environment allowed for greater control, thereby facilitating the extraction of strawberry features and subsequent analysis(Zhang et al., 2016; Indrabayu et al., 2019; Su et al., 2021). Compared to the unstructured outdoor environment, the complexity of lighting, background similarity to fruit, overlapping fruit, and fruit occlusion by plants are some of the uncertain factors that can pose a challenge (Yu et al., 2019; Pérez-Borrero et al., 2020). The presence of these phenomena poses a challenge in precisely segmenting the target fruit from the surrounding environment, thereby impacting the subsequent research work. The significant improvement of AP in **Table 4** is specifically reflected in the model’s miss rate of strawberries and the integrity of the segmentation mask. Thanks to the unique architecture of self-calibrated convolution, the model shows the potential of greater adaptability in the face of complex field environments.

**Table 11:** Comparison of different ripeness identification methods.

Source	Classes	Environment	Model	Results
Zhang et al. (2016)	3	Laboratory	SVM	Accuracy: over 85%
Habaragamuwa et al. (2018)	2	Field	DCNN	AP: 88.03%, 77.21%
Indrabayu et al. (2019)	3	Laboratory	SVM	Accuracy: 85.64%
Shao et al. (2020)	3	Laboratory, Field	PLS-DA, LS-SVM	Accuracy: 91.7% ~ 96.7%
Su et al. (2021)	4	Laboratory	1D ResNet, 3D ResNet	Accuracy: 86.03%, 85.29%
Fan et al. (2022)	4	Field	YOLOv5	Accuracy: over 90%
Raj et al. (2022)	3	Laboratory, Field	SVM	Accuracy: over 98%, 71%
Ours	6	Field	Mask R-CNN,SVM	Accuracy: 86.6%

Strawberries undergo a brief veraison period and mature rapidly. By utilizing a more comprehensive categorization of ripeness stages, fruit farmers can obtain precise information on fruit growth, enabling them to efficiently seize crop management opportunities such as topdressing and harvesting. In this study, strawberries were categorized into six ripeness levels, providing more comprehensive information on their ripeness than previous studies. Due to the large similarity between some categories (such as Turning-1 and Turning-2), it is difficult for the classifier to distinguish them, which eventually leads to a decrease in the overall accuracy (**Table 10**). This phenomenon is also evident in other studies on fruit ripeness. (Saranya et al., 2021; Chen et al., 2022). Categorizing strawberries into 2 to 3 ripeness levels enhances the distinctiveness of their characteristics, facilitating the classifier’s judgment and contributing to the high accuracy achieved in previous studies (Habaragamuwa et al., 2018; Shao et al., 2020; Raj et al., 2022). However, the rough ripeness classification will make the strawberry interval span larger. This often leads to missed opportunities for timely topdressing during the intermediate stages of ripeness and the optimal timing for harvest under various sales patterns towards the end of ripeness. We devised a color feature extraction method that incorporates region segmentation, along with a classifier tailored to the feature data, resulting in precise classification of strawberries into six ripeness levels. The method we proposed not only enables the completion of multi-category ripeness distinction, but also ensures high accuracy. This provides important technical support for the precise harvesting operation of strawberries.

## 5 Conclusion

This study presents a fine recognition method for assessing strawberry ripeness, with the objective of addressing the current issue of coarse classification and emphasizing indoor experimental investigations. It can provide more accurate decision support for strawberry harvest management. The achievement of fine recognition of strawberry ripeness in the field involves three stages. The first stage is to detect and segment strawberries from images with a deep learning model. We added self-calibrated convolutions to Mask R-CNN to improve the network segmentation effect, and the final AP and AP.50 were 0.937 and 0.979, respectively. The second stage is strawberry color feature extraction. Firstly, to extract relevant features, the change trend of feature values with ripeness was analyzed, leading to the selection of channels B, G, L, a, and S for feature extraction. Subsequently, the strawberry was divided into four sub-regions, and the feature values of each region were individually extracted under the aforementioned color channels. The third stage is ripeness classification. The feature values were input into different classification models for ripeness classification, and finally achieved the best results in the SVM classifier. The classification accuracy of SVM is 0.850 under single channel a and 0.866 under combined channel BGLaS. Through additional experiments, it was observed that sub-regions R_1_ and R_2_ primarily play a role in identifying strawberry ripeness in the White, Breaking, and Turning-1 stages. On the other hand, sub-regions R_3_ and R_4_ demonstrated significant contributions in identifying strawberry ripeness in the Turning-2, Ripe, and Full ripe stages.

In summary, the incorporation of self-calibrated convolutions enhances the model’s robustness in field environments, leading to improved segmentation outcomes for strawberries. Additionally, the color feature extraction method based on region segmentation effectively captures the distinctive feature information among strawberries of varying ripeness levels, thus enhancing the classifier’s ability to differentiate between strawberries at different stages of ripeness. The research findings demonstrate that this method can accurately identify multiple levels of ripeness for strawberries in field conditions, thereby providing more effective guidance for strawberry harvest management.

## Data availability statement

The original contributions presented in the study are included in the article/supplementary material. Further inquiries can be directed to the corresponding author.

## Author contributions

CT designed the experiment, conducted data analysis, and wrote the manuscript. XW guided the experiment, provided research ideas, and improved the quality of the manuscript content. XN enhanced the logic and presentation of the Introduction. YeL and YiL processed experimental data and revised figure descriptions. DC and XM contributed to the revision of the manuscript content. SW reviewed and guided the manuscript. All authors contributed to the article and approved it for publication.
